# A comparison of microRNA expression profiles from splenic hemangiosarcoma, splenic nodular hyperplasia, and normal spleens of dogs

**DOI:** 10.1186/s12917-016-0903-5

**Published:** 2016-12-03

**Authors:** Janet A. Grimes, Nripesh Prasad, Shawn Levy, Russell Cattley, Stephanie Lindley, Harry W. Boothe, Ralph A. Henderson, Bruce F. Smith

**Affiliations:** 1 Department of Clinical Sciences, Auburn University College of Veterinary Medicine, Auburn University, Auburn, AL USA; 2 Genomics Services Laboratory, HudsonAlpha Institute for Biotechnology, Huntsville, AL USA; 3 Department of Pathobiology, Auburn University College of Veterinary Medicine, Auburn University, Auburn, AL USA; 4 Scott Ritchey Research Center, Auburn University College of Veterinary Medicine, Auburn University, Auburn, AL USA; 5 Department of Small Animal Medicine and Surgery, College of Veterinary Medicine, University of Georgia, 2200 College Station Road, Athens, GA 30602 USA

**Keywords:** Splenic mass, Hemangiosarcoma, Canine, MicroRNA, RNA-sequencing, Angiogenesis

## Abstract

**Background:**

Splenic masses are common in older dogs; yet diagnosis preceding splenectomy and histopathology remains elusive. MicroRNAs (miRNAs) are short, non-coding RNAs that play a role in post-transcriptional regulation, and differential expression of miRNAs between normal and tumor tissue has been used to diagnose neoplastic diseases. The objective of this study was to determine differential expression of miRNAs by use of RNA-sequencing in canine spleens that were histologically confirmed as hemangiosarcoma, nodular hyperplasia, or normal.

**Results:**

Twenty-two miRNAs were found to be differentially expressed in hemangiosarcoma samples (4 between hemangiosarcoma and both nodular hyperplasia and normal spleen and 18 between hemangiosarcoma and normal spleen only). In particular, mir-26a, mir-126, mir-139, mir-140, mir-150, mir-203, mir-424, mir-503, mir-505, mir-542, mir-30e, mir-33b, mir-365, mir-758, mir-22, and mir-452 are of interest in the pathogenesis of hemangiosarcoma.

**Conclusions:**

Findings of this study confirm the hypothesis that miRNA expression profiles are different between canine splenic hemangiosarcoma, nodular hyperplasia, and normal spleens. A large portion of the differentially expressed miRNAs have roles in angiogenesis, with an additional group of miRNAs being dysregulated in vascular disease processes. Two other miRNAs have been implicated in cancer pathways such as PTEN and cell cycle checkpoints. The finding of multiple miRNAs with roles in angiogenesis and vascular disease is important, as hemangiosarcoma is a tumor of endothelial cells, which are driven by angiogenic stimuli. This study shows that miRNA dysregulation is a potential player in the pathogenesis of canine splenic hemangiosarcoma.

## Background

Splenic masses are common in older dogs and may be malignant, benign, or non-neoplastic; yet diagnosis preceding splenectomy and histopathology remains elusive. Several studies have reported approximately 70% of dogs with non-traumatic hemoperitoneum had hemangiosarcoma [1–3]. Hemoperitoneum is reported in 63–80% of dogs with hemangiosarcoma, compared with only 30% of dogs with benign splenic masses [4, 5]. This has led to the ‘double 2/3 rule,’ which is currently used to give owners a prediction of the odds of each of the possibilities [6]. According to this rule, approximately 2/3 of splenic masses are malignant, and of those that are malignant, 2/3 are hemangiosarcoma. Other malignant splenic masses include various sarcomas, lymphoma, and histiocytic sarcoma [1, 7]. Benign and non-malignant splenic conditions include hemangioma, nodular hyperplasia, formerly classified as a subset of fibrohistiocytic nodules, and hematoma [1, 8].

Many studies have attempted to identify repeatable measures or other techniques that might distinguish malignant from benign masses [5, 9–14]. For instance, mass-to-splenic volume ratio and splenic weight as a percentage of body weight have been used to differentiate malignant from benign splenic lesions, with hemangiosarcoma masses being smaller in both categories [5]. However, these values can only be calculated after splenectomy, and splenic size can change due to contraction or engorgement in response to medications or hemoperitoneum. Diagnostic imaging has been evaluated for its ability to differentiate malignant from benign lesions with contrast harmonic ultrasound, CT, and MRI showing promise [9–11]. Such modalities may differentiate malignant from benign lesions but do not diagnose a specific disease process. Prognosis and survival times between various malignancies can be quite varied and availability of these treatment modalities is limited and may be cost prohibitive [1, 12, 15]. While splenic aspirates may be beneficial for certain neoplasms such as lymphosarcoma, they usually fail to aid in the diagnosis of many splenic tumors due to blood contamination and poor exfoliation. Also, some clinicians recommend not aspirating the spleen in suspected cases of hemangiosarcoma due to risk of tumor rupture and seeding of the tumor into the abdomen [5, 6]. Testing of blood with multi-parameter flow cytometry and measuring levels of vascular endothelial growth factor and thymidine kinase have been evaluated, but have not been found to be definitive diagnostic tools [12–14]. It is clear that additional work needs to be done to develop a minimally invasive pre-surgical diagnostic test to differentiate hemangiosarcoma from other splenic masses.

Hemangiosarcoma, a tumor of vascular endothelial origin, is the most common splenic tumor, and the prognosis is poor: dogs that undergo surgery alone as a treatment for splenic hemangiosarcoma have a median survival time of three months; this extends to six months if chemotherapy is used in conjunction with surgery [6]. The decision to proceed with surgery can be difficult for owners because although there are rare long-term survivors, median survival times are typically short, and currently there is no ability to give a definitive diagnosis prior to surgery and histopathology.

MicroRNAs (miRNAs) are 18–25 nucleotide, single stranded, non-coding RNAs that play a role in post-transcriptional regulation [16–18]. MicroRNAs inhibit expression of target genes by binding to the 3’ untranslated region of certain messenger RNAs (mRNAs) [16, 17]. MicroRNAs have a role in cell growth, cell differentiation, apoptosis, and oncogenesis [17]. Expression profiles give information on the identities and quantities of particular miRNAs within a given tissue; such profiles are consistent between like-tissue samples [19]. MicroRNAs in tumor samples have been used to diagnose tumors, provide prognostic information, and aid in targeted treatments in human medicine [18–22]. Many tumor types have been evaluated for differential miRNA expression, including ovarian carcinomas, breast cancer, cervical cancer, non-small cell lung cancer, leukemias, colorectal tumors, squamous cell carcinoma, and hepatocellular carcinoma [21–28]. Use of miRNAs in support of other diagnostic methods is currently in its infancy, with miRNA signatures having been developed in people to distinguish melanoma and metastatic breast cancer from healthy controls and higher risk groups in breast cancer and prostate cancer [29–32]. There are few reports of miRNA involvement in cancer of veterinary patients, but interest in this area will likely increase with the rapid growth of this topic in human medicine [33–35].

MicroRNAs have excellent stability in serum, and miRNAs representative of cancer tissue have been identified in the circulation of patients with cancer [20]. Such identification allows for the potential to develop a noninvasive diagnostic test to diagnose cancers, without having to obtain a tissue sample of the tumor of interest. The objective of this study was to identify and compare expression profiles of miRNAs from canine splenic hemangiosarcoma, splenic nodular hyperplasia, and normal splenic tissues using RNA-sequencing. We hypothesized that there would be differences in miRNA expression among the three groups. This work is the first step in determining altered miRNA expression in canine splenic masses. Once altered miRNA expression has been identified in the tissues, future studies can be performed to evaluate these same altered miRNAs in the serum of patients with splenic masses. The end goal of this research is to develop a blood-based diagnostic test to determine the nature of canine splenic masses. Ultimately, this work may also provide insight into pathways that are dysregulated in hemangiosarcoma, allowing a better understanding of both tumorigenesis and potential therapies.

## Methods

### Sample collection

Splenic mass samples: Samples were collected from spleens removed from client-owned animals undergoing splenectomy for a splenic mass. After removal of the spleen, the mass was trimmed to obtain two samples: one for the study and an adjacent piece of tissue for histopathologic evaluation to confirm a diagnosis and ensure that representative tissue was present in the sample. Samples to be used for the study were flash frozen with liquid nitrogen within 30 min of splenectomy and stored in a −80 °C freezer until further use. Only masses confirmed to contain tissue from hemangiosarcoma or nodular hyperplasia were used for the study. Five samples in each category (hemangiosarcoma and nodular hyperplasia) were collected.

Normal spleen samples: Archived fresh frozen tissue samples were utilized to analyze normal splenic tissue. Samples were collected within 30 min of splenectomy, flash frozen in liquid nitrogen, and stored in a −80 °C freezer. Histopathology of adjacent tissue performed at the time of sample collection confirmed these five spleens to be normal.

### Histopathology

Tissues (splenic masses and normal spleens) were trimmed and fixed in 10% neutral-buffered formalin for 24–72 h prior to processing by paraffin impregnation. Sections approximately 4–5 microns thick were prepared by microtomy, mounted on glass slides, deparaffinized, and stained with hematoxylin and eosin prior to applying glass coverslips. Each slide was evaluated by light microscopy for diagnosis by a board-certified (ACVP) pathologist. Cases of hemangiosarcoma were confirmed by demonstration of CD31 by immunohistochemistry (Dako, Denmark). Sections were mounted onto slides, deparaffinized, heat-treated for antigen retrieval, and labeled with CD31 using FLEX monoclonal mouse anti-human CD31 clone JC70A visualized by peroxidase-mediated oxidation of diaminobenzidine (EnVision FLEX+ Mouse High pH Link system, Dako, Denmark). Slides were coverslipped, counterstained with hematoxylin, and examined by light microscopy.

### RNA isolation

The Qiagen miRNeasy kit (Qiagen Inc., Valencia, CA, USA) was utilized to extract RNA from the frozen tissue sections. Extraction was performed according to manufacturer protocol using the Bullet Blender (Next Advance Inc., Averill Park, NY, USA) for homogenization, and one modification to the protocol, where the Buffer RWT step was repeated for a second time. The NanoDrop (ThermoScientific, Wilmington, DE, USA) was used to confirm an appropriate 260/280 and 260/230 ratio for the sample (>1.8 in each case).

### RNA Sequencing and smRNA library prep protocol

RNA samples were sent to the Genomic Services Laboratory at the HudsonAlpha Institute for Biotechnology for miRNA-sequencing analysis. NEBNext® Small RNA Library Prep Set for Illumina® (New England BioLabs Inc., Ipswich, MA, USA) was utilized. Three prime adapters were ligated to total input RNA followed by hybridization of multiplex SR RT primers and ligation of multiplex 5` SR adapters. Reverse Transcription (RT) was done using SuperScript III RT (Life Technologies, Grand Island, NY, USA) for 1 h at 50 °C. Immediately after RT reaction, indexed primers were added to uniquely barcode each sample and PCR amplification was done for 12 cycles using LongAmp Taq 2X master mix. Post PCR material was then purified using QIAquick PCR purification kit (Qiagen Inc., Valencia, CA, USA). Post PCR yield and concentration of the prepared libraries was assessed using Qubit® 2.0 Fluorometer and DNA 1000 chip on Agilent 2100 Bioanalyzer.

Size selection of small RNA libraries with a target size range of 140 base pairs was done on a 3% agarose gel using Pipin prep instrument (Sage Science, Boston, MA, USA). Accurate quantification for sequencing applications was performed using the qPCR-based KAPA Biosystems Library Quantification kit. Each library was diluted to a final concentration of 12.5 nM and pooled equimolar prior to clustering. Cluster generation was carried out on a cBot v1.4.36.0 using Illumina's Truseq Single Read (SR) Cluster Kit v3.0. Single End (SE) sequencing was performed on an Illumina HiSeq2000, running HiSeq Control Software (HCS) v1.5.15.1, using a 50 cycle TruSeq SBS HS v3 reagent kit. The clustered flowcells were sequenced for 56 cycles, consisting of a 50 cycle read, followed by a 6 cycle index read. Image analysis and base calling was performed using the standard Illumina Pipeline consisting of Real Time Analysis (RTA) version v1.13 and demultiplexed using bcl2fastq converter with default settings.

### Analysis

Post-processing of the sequencing reads from miRNA-sequencing experiments from each sample was performed as per unique in-house pipelines. Briefly, quality control checks on raw sequence data from each sample was performed using FastQC (Babraham Bioinformatics, London, UK). Raw reads were imported on a commercial data analysis platform CLCbio (Qiagen Inc., Valencia, CA, USA). Adapter trimming (GTGACTGGAGTTCAGACGTGTGCTCTTCCGATCT) was done to remove ligated adapters from the 3' end of the sequenced reads with only one mismatch allowed; poorly aligned 3' ends were also trimmed. Sequences shorter than 15 nucleotides length were excluded from further analysis. Trimmed reads with low qualities (base quality score less than 30, alignment score less than 95, mapping quality less than 40) were removed. Filtered reads were used to extract and count the small RNA that were annotated with miRNAs from the miRBase release 20 database [36, 37].

The quantification operation carried out measurement at both the gene level and at the active region level. Active region quantification considered only reads whose 5' end matched the 5' end of the mature miRNA annotation. Samples were grouped as patient and control identifiers, and differential expression of miRNA was calculated on the basis of their fold change observed between individual patients and averaged control samples. The p-value of differentially expressed miRNAs was estimated by implementing t-tests with Benjamini Hochberg false discovery rate corrections of 0.05 [38].

## Results

**Fig. 1:**
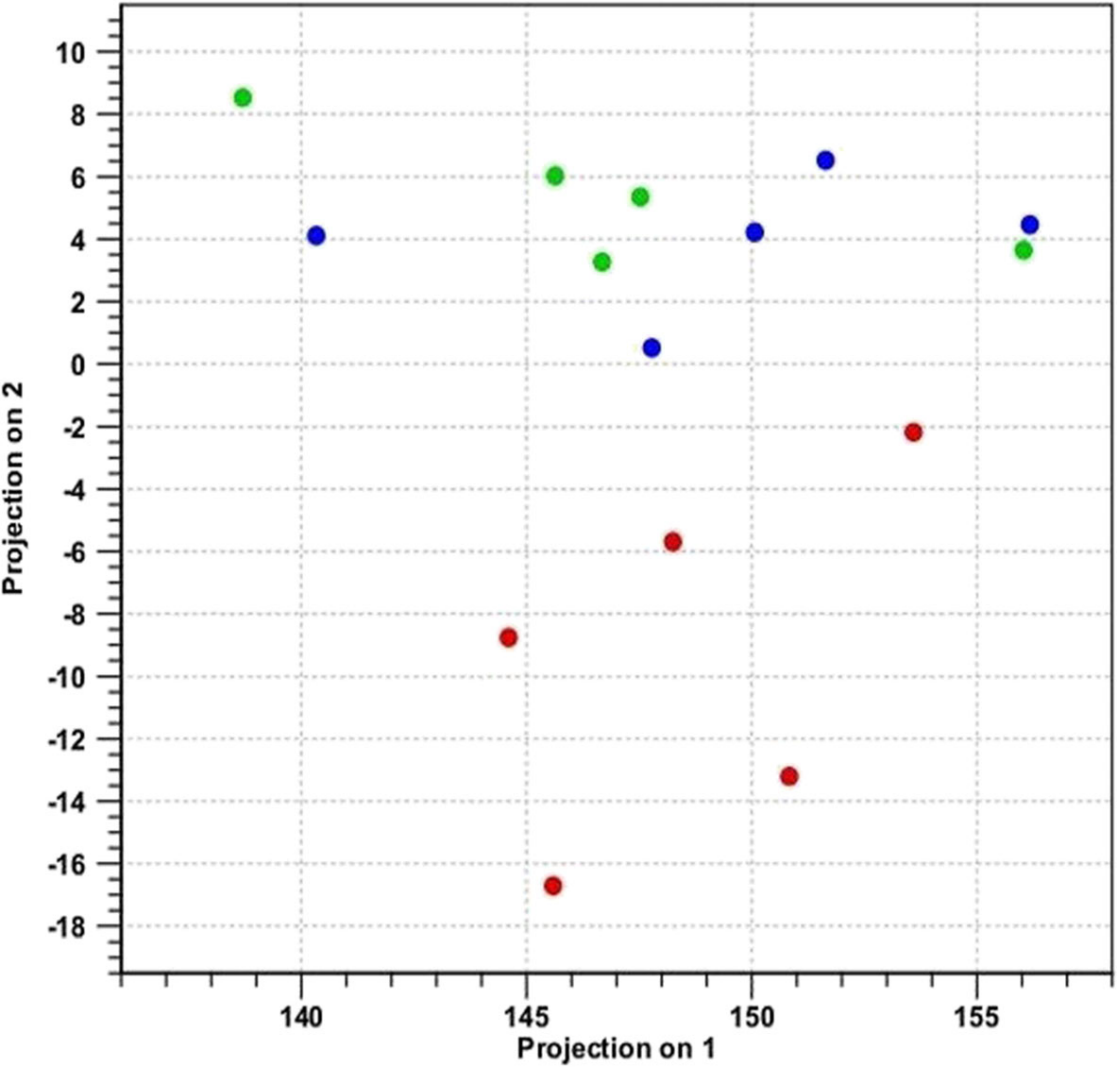
Principal component analysis of hemangiosarcoma, nodular hyperplasia, and normal spleen samples. The hemangiosarcoma samples (*red*) showed differential expression from both nodular hyperplasia (*blue*) and normal spleen (*green*) samples. The axes correspond to principal component 1 (x-axis) and principal component 2 (y-axis)

**Fig. 2:**
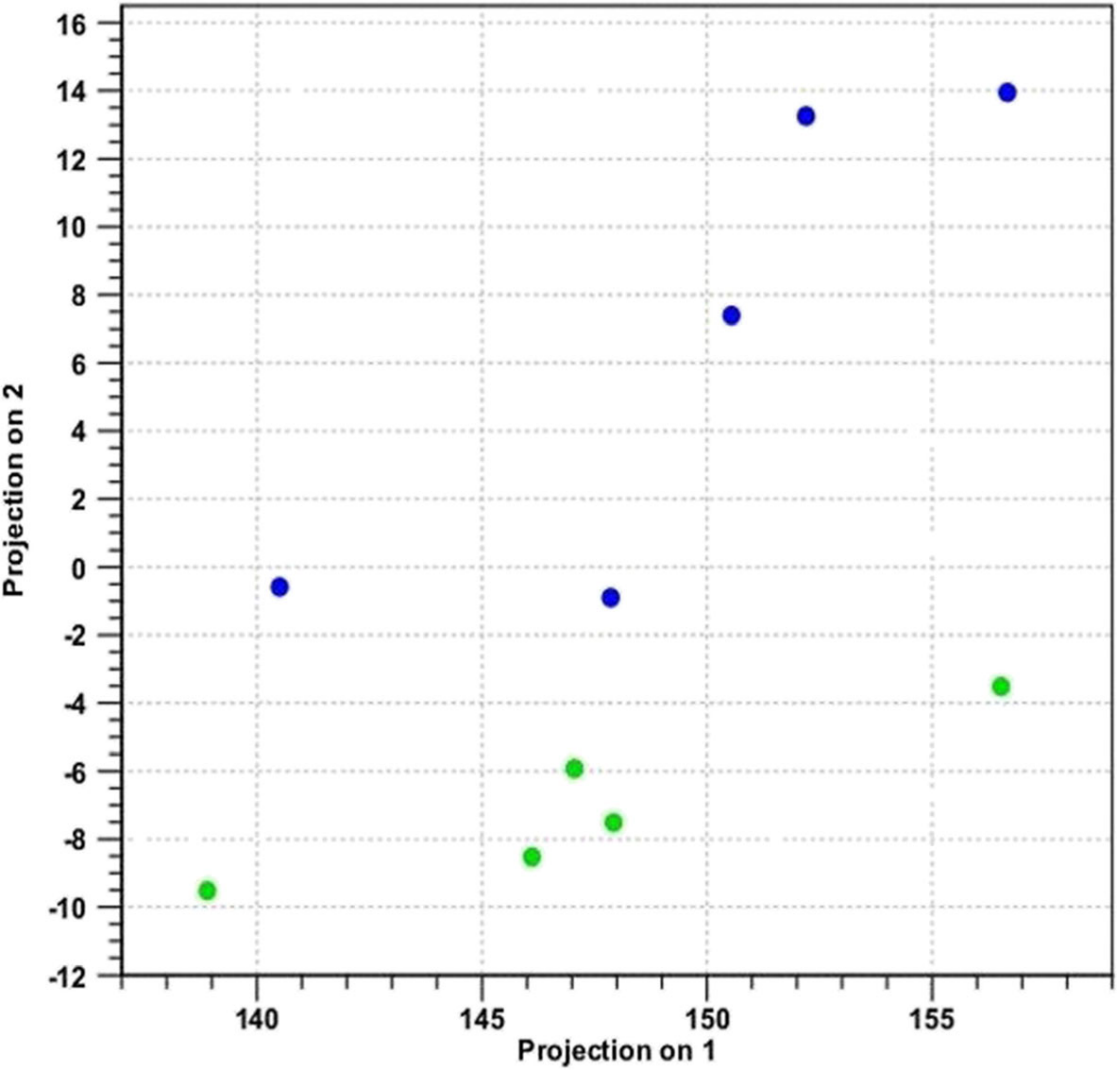
Principal component analysis of nodular hyperplasia and normal spleen samples. The nodular hyperplasia samples (*blue*) showed differential expression from normal spleen samples (*green*). The axes correspond to principal component 1 (x-axis) and principal component 2 (y-axis)

**Fig. 3:**
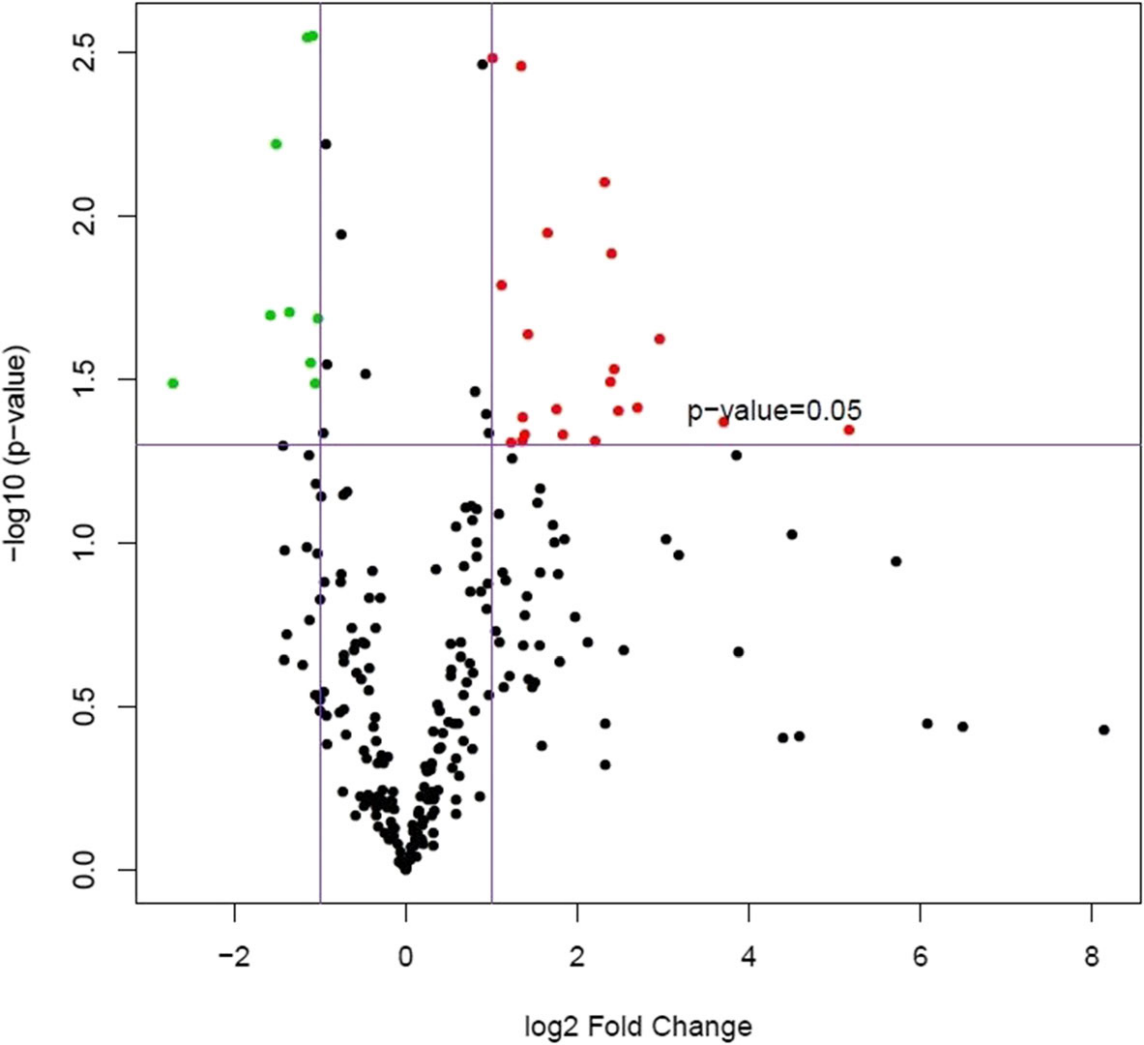
Volcano plot showing significantly overexpressed (*red*) and significantly underexpressed (*green*) miRNAs between hemangiosarcoma and normal spleen. The axes correspond to log_2_ (fold change) (x-axis) and -log_10_ (*p*-value) (y-axis)

**Fig. 4:**
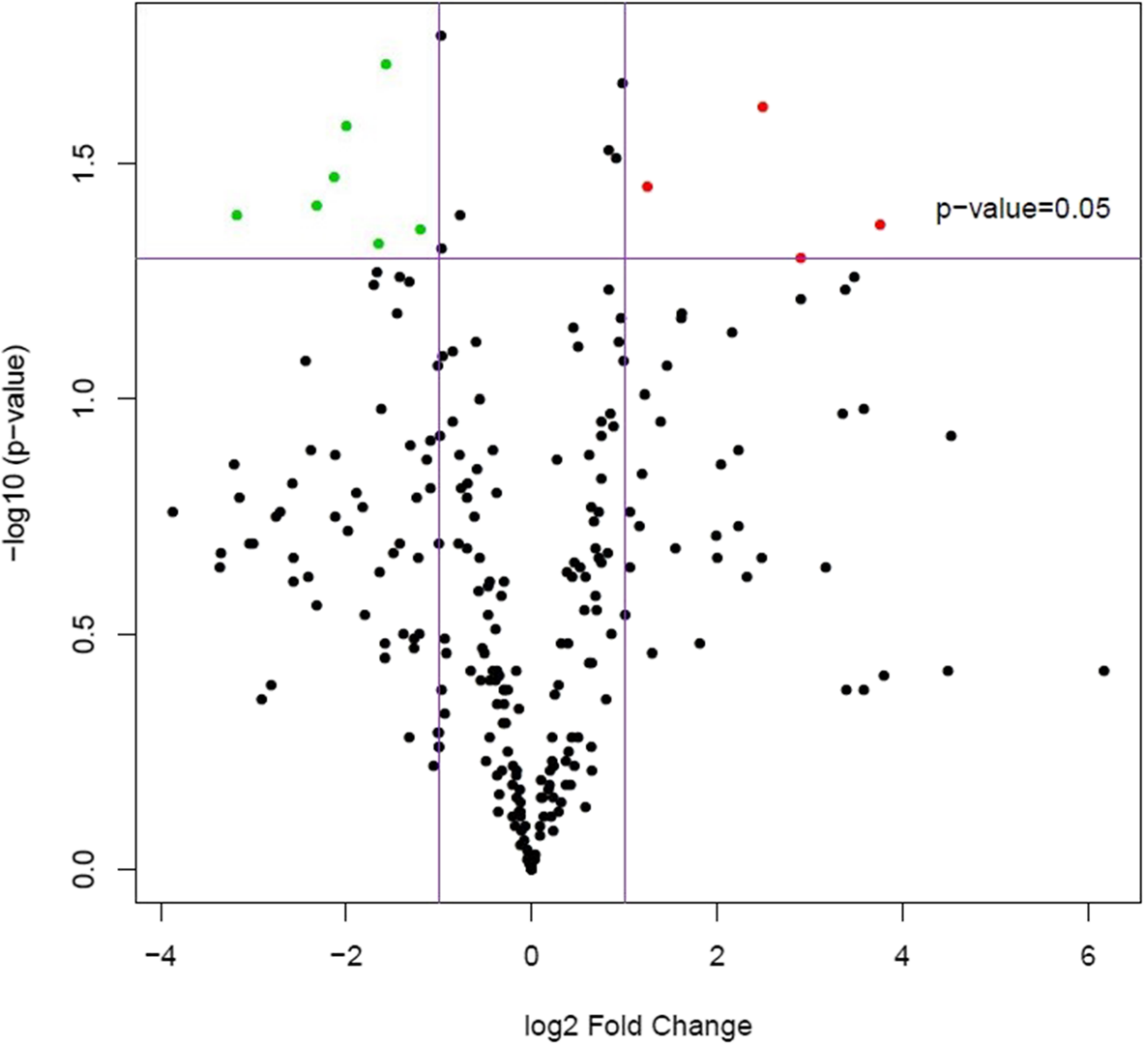
Volcano plot showing significantly overexpressed (*red*) and significantly underexpressed (*green*) miRNAs between hemangiosarcoma and nodular hyperplasia. The axes correspond to log_2_ (fold change) (x-axis) and -log_10_ (*p*-value) (y-axis)

**Fig. 5:**
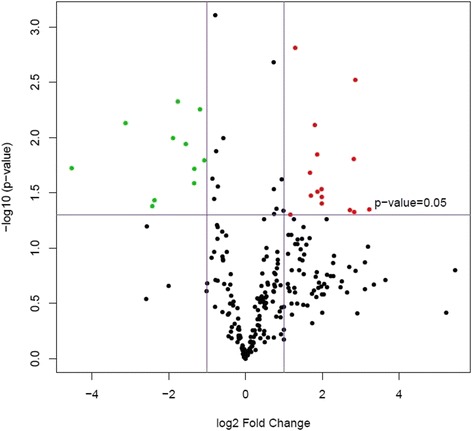
Volcano plot showing significantly overexpressed (*red*) and significantly underexpressed (*green*) miRNAs between nodular hyperplasia and normal spleen. The axes correspond to log_2_ (fold change) (x-axis) and -log_10_ (*p*-value) (y-axis)

Principal component analysis was performed compiling the miRNA data from all five samples within each group. This revealed hemangiosarcoma samples grouped independently from nodular hyperplasia and normal spleen, indicating hemangiosarcoma samples were distinctly different than the other two categories (Fig. 1). When hemangiosarcoma samples were removed from the analysis, nodular hyperplasia and normal spleen samples also showed differential expression, indicating that it may be possible to distinguish between these two conditions with further analyses (Fig. 2). Volcano plots were created of the comparison groups highlighting miRNAs that were significantly over or underexpressed between the groups. Significant over and underexpression of various miRNAs was found for each of the three groups: hemangiosarcoma compared to normal spleen (Fig. 3), hemangiosarcoma compared to nodular hyperplasia (Fig. 4), and nodular hyperplasia compared to normal spleen (Fig. 5).

**Fig. 6:**
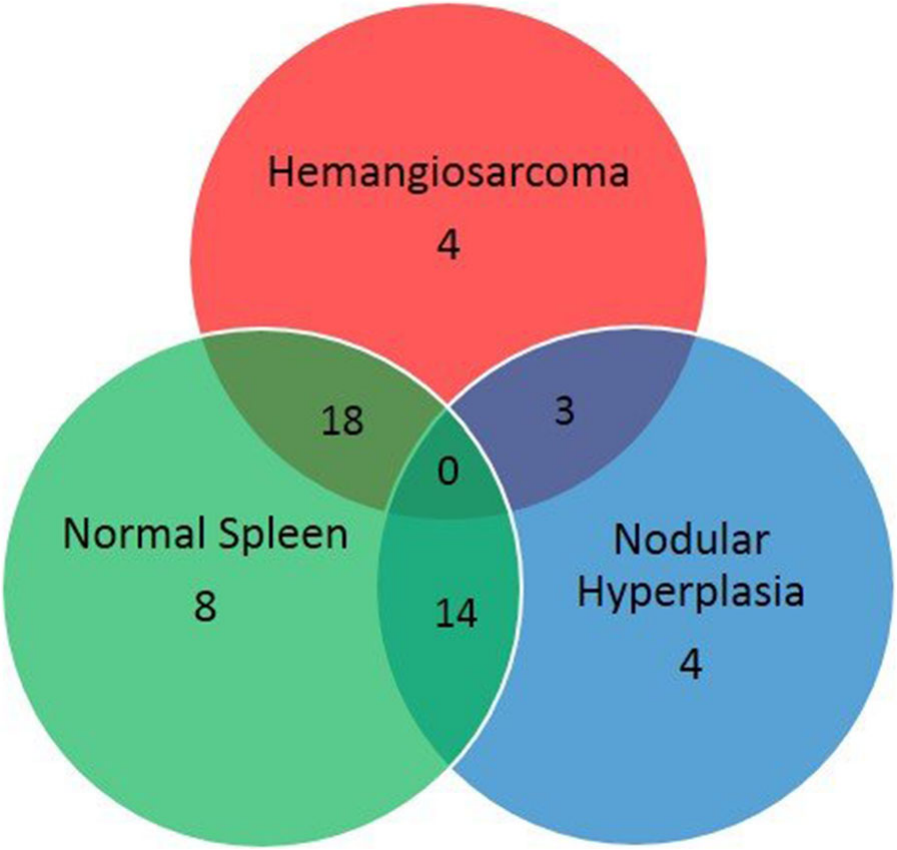
Venn diagram demonstrating miRNAs differentially expressed between hemangiosarcoma, nodular hyperplasia, and normal spleen (fold change ≥ ± 2, significance set a *p* < 0.05). Eighteen miRNAs were differentially expressed solely between hemangiosarcoma and normal spleen samples. Fourteen miRNAs were differentially expressed solely between nodular hyperplasia and normal spleen samples. Three miRNAs were differentially expressed solely between hemangiosarcoma and nodular hyperplasia samples. Four miRNAs were determined to be potential markers of hemangiosarcoma as they were differentially expressed between hemangiosarcoma and nodular hyperplasia samples and also hemangiosarcoma and normal spleen samples. Four miRNAs were determined to be potential markers of nodular hyperplasia as they were differentially expressed between hemangiosarcoma and nodular hyperplasia samples and also nodular hyperplasia and normal spleen samples. Eight miRNAs were determined to be potential markers of normal splenic tissue as they were differentially expressed between hemangiosarcoma and normal spleen samples and also nodular hyperplasia and normal spleen samples

Individual microRNA results were evaluated, significance was set at *p* < 0.05, and data were limited to microRNAs with a fold change ≥ ± 2. With these criteria, 51 unique miRNAs were found to be differentially expressed across the three groups (Fig. 6), with 4 miRNAs being potential candidates specific to hemangiosarcoma (Table 1) and 18 being differentially expressed between hemangiosarcoma and normal spleen only (Table 2). No miRNAs were significantly differentially expressed between all of the three possible pairings.

**Table 1:** MiRNAs significantly differentially expressed between hemangiosarcoma and both nodular hyperplasia and normal spleen^a^

MicroRNA	Fold Change (HSA vs. NS)	*p*-value (HSA vs. NS)	Fold Change (HSA vs. NH)	*p*-value (HSA vs. NH)	HSA (Means)	NH (Means)	NS (Means)
mir-126	3.382336	0.038943	5.614515	0.023733	40134.8	7148.4	11866
mir-150	−2.84562	0.006069	−9.05708	0.040751	3097.6	28055.2	8814.6
mir-203	−2.56436	0.01982	−2.29043	0.043515	60.6	138.8	155.4
mir-452	13.06709	0.042741	13.54305	0.042579	818	60.4	62.6

^a^ fold change ≥ ± 2 and significance set a *p* < 0.05

*HSA* hemangiosarcoma, *NS* normal spleen, *NH* nodular hyperplasia

**Table 2:** MiRNAs significantly differentially expressed between hemangiosarcoma and normal spleen only^a^

MicroRNA	Fold Change	p-value	Hemangiosarcoma (Means)	Normal Spleen (Means)
mir-139	3.554455	0.046672	71.8	20.2
mir-140	−2.21531	0.002865	12837.4	28438.8
mir-188	2.539683	0.003478	192	75.6
mir-193a	5.277433	0.013137	509.8	96.6
mir-22	2.010375	0.003289	65029.2	32346.8
mir-26a-2//mir-26a-1	−2.03635	0.020626	118478.2	241263.2
mir-301b	2.34	0.049404	46.8	20
mir-30e	−2.1239	0.002838	2931.4	6226
mir-33b	2.569892	0.041145	47.8	18.6
mir-365-2//mir-365-1	4.615929	0.048929	1043.2	226
mir-424	4.978495	0.007939	92.6	18.6
mir-450a	7.765751	0.023887	3673.2	473
mir-450b	2.68626	0.023021	3519	1310
mir-503	5.391026	0.029408	168.2	31.2
mir-505	2.169014	0.016269	30.8	14.2
mir-542	5.563845	0.039428	2326.8	418.2
mir-758	3.142857	0.011275	4.4	1.4
mir-876	6.5	0.038656	2.6	0.4

^a^ fold change ≥ ± 2 and significance set a *p* < 0.05

### Hemangiosarcoma compared to both normal spleen and nodular hyperplasia

Four miRNAs were significantly different between hemangiosarcoma samples and both normal spleens and spleens with nodular hyperplasia, indicating these miRNAs may be markers specific for hemangiosarcoma (Table 1). Of these miRNAs, two were significantly overexpressed (mir-126, mir-452) and two significantly underexpressed (mir-150, mir-203) in hemangiosarcoma samples compared to both normal spleens and spleens with nodular hyperplasia.

### Hemangiosarcoma compared to normal spleen

Eighteen miRNAs were differentially expressed between hemangiosarcoma and normal spleen only (without also playing a role in nodular hyperplasia samples), with 15 being significantly overexpressed in hemangiosarcoma samples and three being underexpressed (Table 2).

## Discussion

The results of this study confirm the hypothesis that miRNAs are differentially expressed in the tissues of canines with splenic hemangiosarcoma, splenic nodular hyperplasia, and normal spleens.

Four miRNAs were identified as potential markers of hemangiosarcoma, as they were differentially expressed in hemangiosarcoma samples compared to both normal spleen and nodular hyperplasia samples: mir-126, mir-150, mir-203, and mir-452. Mir-126 and mir-452 were significantly overexpressed in hemangiosarcoma samples, while mir-150 and mir-203 were significantly underexpressed in hemangiosarcoma samples compared to normal spleen and nodular hyperplasia samples. Three of these miRNAs, mir-126, mir-150, and mir-203 have previously been found to have roles in angiogenesis [39–47]. Previous reviews have confirmed that mir-126 is expressed in higher numbers in vascular tissues such as heart, liver, and lung and also in endothelial cell lineage cells [39, 43, 48]. Additional work has shown that mir-126 levels are increased in endothelial precursor cells, which are the cells of origin of hemangiosarcoma, explaining their upregulation in this particular tumor type [12, 40–43]. Mir-126 can behave in both pro- and anti-angiogenic ways, but is pro-angiogenic in endothelial precursor cells and actively proliferating and migrating endothelial cells [41]. Mir-126 enhances angiogenesis by increasing VEGF expression through its targeting of the PI3K regulatory subunit 2 (p85β) [39, 40, 43, 49]. Dogs with hemangiosarcoma have higher plasma VEGF levels than healthy controls, which correlates with the findings of increased mir-126 expression in hemangiosarcoma samples [13]. The PI3K pathway has been previously implicated in canine hemangiosarcoma as well, with mutations in PTEN leading to increased phosphorylated Akt [50]. Mir-126 may be acting in concert with other mediators to influence this pathway, leading to increased VEGF production and a pro-survival state. Mir-126 also targets regulator of G-protein signaling (RGS16) which inhibits CXCR4, an important protein in angiogenesis [41, 51]. When CXCR4 is activated, both circulating hematopoietic stem cells and prostate cancer cells have increased endothelial cell adhesion and transendothelial migration, indicating this pathway may direct metastasis [52, 53]. Mir-150 also plays a role in regulation of CXCR4, with decreased expression of mir-150 (as was seen in the hemangiosarcoma samples) leading to increased expression of CXCR4 protein [45, 46]. VEGF has also been confirmed to be a direct target of mir-150, and downregulation of mir-150 led to increased VEGF expression in brain microvascular endothelial cells, leading to increased proliferation and migration of these cells [44]. Mir-203, which was downregulated in the hemangiosarcoma samples, has been shown to be a tumor suppressor that targets VEGFA, with increased expression of mir-203 leading to suppression of VEGFA in cervical cancer [47]. Although mir-452 has not been previously associated with angiogenic-specific pathways, it has been shown to target cyclin-dependent kinase inhibitor 1B, an inhibitor of the cell cycle checkpoint from G1 to S [54]. Hepatocellular carcinoma cells significantly overexpress mir-452, leading to increased cell invasion and migration and inhibition of apoptosis [54]. This miRNA was overexpressed by 13-fold in hemangiosarcoma samples compared to both nodular hyperplasia and normal spleen samples, indicating dysregulation of the cell cycle checkpoints may be a key player in the transition to hemangiosarcoma.

The 18 miRNAs significantly different between hemangiosarcoma and normal spleen only were further investigated for potential downstream targets. Seven of these, mir-139, mir-140, mir-26a, mir-424, mir-503, mir-505, and mir-542 have been shown to be involved in angiogenesis [55–63]. Although mir-139 has been reported to act as a tumor suppressor in most studies, its role in angiogenesis is becoming clearer [55, 56, 64, 65]. Mir-139 was found to increase cancer endothelial cell migration and promote vessel formation in pancreatic cancer [55]. Mir-139 was also found to negatively regulate CXCR4, playing a role in tightly regulating angiogenesis to prevent over-activation of endothelial cells [56]. It is possible that mir-139 is upregulated in response to the increased CXCR4 levels associated with mir-126 and mir-150 overexpression. Both mir-140 and mir-26a directly target VEGFA to repress its expression [57, 58]. These 2 miRNAs were underexpressed in the hemangiosarcoma samples compared to normal spleen, which fits with previous findings of increased VEGF expression in patients with hemangiosarcoma [13]. Mir-424 was found to be increased in tissues undergoing vascular remodeling after hypoxia, resulting in increased cell migration, and blockade of mir-424 led to decreased proliferation and vascular tube formation [59]. Another group found a contradictory function, in that mir-424 regulated VEGF and bFGF signaling by reducing expression of receptors for those cytokines and increased expression of mir-424 led to reduced proliferation and migration of endothelial cells [60]. This group also found that VEGF and bFGF had stimulatory effects on mir-424 expression, indicating that increased levels of VEGF, as seen in hemangiosarcoma, may have led to the finding of mir-424 being overexpressed, participating in a negative feedback loop [14, 60]. While it remains clear that mir-424 plays a role in angiogenesis, further studies are warranted to evaluate its specific role in canine hemangiosarcoma. Mir-503 is transcribed with mir-424 due to their close proximity, and mir-503 has also been shown to be anti-angiogenic by targeting VEGFA [61–63]. Mir-505, which was increased in the hemangiosarcoma samples, has been shown to decrease endothelial cell migration and vascular tube formation [66]. One study found that mir-542-3p targeted angiopoietin-2 and acted as an anti-angiogenic signal [67]. Angiogenesis requires a delicate balance of its mediators, and mir-424, mir-503, mir-505, and mir-542 may be overexpressed in these samples due to the effects of the multitude of other miRNAs acting in a pro-angiogenic manner. Vessel formation in hemangiosarcoma should not be strictly compared to normal angiogenesis, as tumor vessels are tortuous and leaky [68]. It is feasible that mixed angiogenic signaling leads to the abnormal vessel formation found in canine hemangiosarcoma. Mir-503 has also been shown to target the PI3K pathway by inhibiting the regulatory subunit, PI3K p85, acting as a tumor suppressor [69]. Again, this finding may be a regulatory negative feedback loop in response to mir-126 overexpression. Further work should be done to evaluate the inter-related roles of these miRNAs.

Mir-22, which was overexpressed in the hemangiosarcoma samples, has been shown to downregulate PTEN, which parallels the previous finding of PTEN inactivation in canine hemangiosarcoma [50, 70–72]. Mir-30e has been shown to be an endogenous miRNA in human microvascular endothelial cells and plays a role in human atherosclerosis by altering differentiation pathways [73–75]. Mir-33b and mir-758, which were overexpressed in the hemangiosarcoma samples, have also been shown to regulate gene expression in human atherosclerotic plaques [76]. Another miRNA, mir-365, which was overexpressed in these samples, has been shown to decrease vascular smooth muscle production in vascular injury repair [77]. It is clear that mir-30e, mir-33b, mir-365, and mir-758 are involved in the vasculature, but their specific role in canine hemangiosarcoma is unclear.

The remaining 6 miRNAs have been previously implicated in neoplasia, but more specific information relating specifically to hemangiosarcoma and/or angiogenesis could not be found [78–83].

Another group has evaluated miRNA expression in canine hemangiosarcoma, specifically looking at mir-214 [84]. This miRNA was found to act as a tumor suppressor by promoting apoptosis, and was downregulated in their samples [84]. Later work by the same group found overexpression of mir-214 in the media of canine hemangiosarcoma and human angiosarcoma cell lines, which contradicted their previous findings of underexpression within the cells themselves [85]. They also found increased expression of mir-214 in the plasma of canine patients with hemangiosarcoma, which decreased after tumor removal [85]. The explanation for the contradictory findings in these studies was that intracellular and extracellular concentrations of miRNAs can be different and because miRNAs can have a multitude of downstream targets, they may act differently depending on their location and the disease state. Mir-214 was not significantly different in expression in the samples reported here. One reason for this may have been the methods used to evaluate for differential expression of miRNA. In the study reported here, RNA-sequencing was used to determine differentially expressed miRNAs, compared to the previously reported studies which used qRT-PCR to evaluate for miRNAs [84, 85]. Both the study reported here and the previously published works had relatively small sample numbers, and evaluation of a larger sample size may help to clarify these confounding results [84, 85]. Despite the lack of agreement in the findings of mir-214, the results reported here agree with the findings of mir-126 reported by the previous group, in which they found overexpression of mir-126 in plasma samples of canine patients with hemangiosarcoma [85]. These previous studies only evaluated mir-214 and mir-126 expression and did not evaluate for other miRNAs, but the finding of mir-126 overexpression, similar to the findings of the current study, is noteworthy. It is important to note that disease stage was not evaluated in the study reported here nor in the previously reported studies evaluating miRNA in canine hemangiosarcoma. This may also help to explain the contradictory findings regarding mir-214, as patients with different disease stages may have different miRNA expression levels. The long-term goal of the study presented here is to identify these dysregulated miRNAs in the circulation of patients with hemangiosarcoma. Mir-126 was overexpressed in these tissue samples, and work by others has shown it to be overexpressed in the serum of canine patients with splenic hemangiosarcoma. The hope is that with additional investigation, other miRNAs that were identified in the current study will be found in the circulation, allowing use of a minimally invasive diagnostic test for canine splenic hemangiosarcoma.

## Conclusions

Results of the current study confirm the hypothesis that miRNAs are significantly differentially expressed between canine splenic hemangiosarcoma, nodular hyperplasia, and normal spleen samples. Ten of the 22 miRNAs dysregulated in hemangiosarcoma samples have been shown to have roles in angiogenesis (mir-26a, mir-126, mir-139, mir-140, mir-150, mir-203, mir-424, mir-503, mir-505, and mir-542). This is of particular importance for this tumor specifically, as it is a tumor of endothelial cells. An additional 4 miRNAs (mir-30e, mir-33b, mir-758, and mir-365) have been shown to be dysregulated in vascular disease processes. Two additional miRNAs (mir-22 and mir-452) have been implicated in cancer pathways, with mir-22 downregulating PTEN, a tumor suppressor that plays a role in hemangiosarcoma, and mir-452 altering cell cycle checkpoints to increase cell replication [54, 70–72]. Although the sample numbers in this study were small, the results point to clear roles of miRNAs in the pathogenesis of hemangiosarcoma via alteration of angiogenic signaling and cancer pathways. Further work needs to be done to evaluate these miRNA in a larger sample size and to elucidate the specific roles these miRNAs play in the angiogenic alterations leading to development of hemangiosarcoma, as the majority of these miRNAs have not been previously implicated in hemangiosarcoma. Further exploration is indicated to identify these miRNA in circulation to allow delineation of a specific miRNA panel that may become useful as a minimally invasive, pre-surgical diagnostic test to differentiate canine splenic hemangiosarcoma from other masses of the spleen.
